# A conceptual framework for measuring community health workforce performance within primary health care systems

**DOI:** 10.1186/s12960-019-0422-0

**Published:** 2019-11-20

**Authors:** Smisha Agarwal, Pooja Sripad, Caroline Johnson, Karen Kirk, Ben Bellows, Joseph Ana, Vince Blaser, Meghan Bruce Kumar, Kathleen Buchholz, Alain Casseus, Nan Chen, Hannah Sarah Faich Dini, Rachel Hoy Deussom, David Jacobstein, Richard Kintu, Nazo Kureshy, Lory Meoli, Lilian Otiso, Neil Pakenham-Walsh, Jérôme Pfaffmann Zambruni, Mallika Raghavan, Ryan Schwarz, John Townsend, Brittney Varpilah, William Weiss, Charlotte E. Warren

**Affiliations:** 1 Population Council, Washington, DC USA; 2 Department of International Health, Johns Hopkins Bloomberg School of Public Health, Baltimore, MD USA; 3 Population Council, New York, NY USA; 4 Healthcare Information For All (HIFA), Oxford, UK; 5 IntraHealth International, Washington, DC USA; 6 Department of International Public Health, Liverpool School of Tropical Medicine, Liverpool, UK; 7 Last Mile Health, New York, USA; 8 Zanmi Lasante, Croix-des-Bouquets, Haiti; 9 UNICEF, New York, USA; 10 HRH2030 Program, Chemonics International, Washington, DC USA; 11 USAID, Washington, DC USA; 12 Pathfinder International, Kampala, Uganda; 13 Nyaya Health Nepal, Kathmandu, Nepal; 14 Brigham and Women’s Hospital, Department of Medicine, Division of Global Health Equity, Boston, MA USA

**Keywords:** Community health worker, Primary health care, Health metrics, Healthcare quality indicators, Health information systems

## Abstract

**Background:**

With the 40th anniversary of the Declaration of Alma-Ata, a global effort is underway to re-focus on strengthening primary health care systems, with emphasis on leveraging community health workers (CHWs) towards the goal of achieving universal health coverage for all. Institutionalizing effective, sustainable community health systems is currently limited by a lack of standard metrics for measuring CHW performance and the systems they work within. Developed through iterative consultations, supported by the Bill & Melinda Gates Foundation and in partnership with USAID and UNICEF, this paper details a framework, list of indicators, and measurement considerations for monitoring CHW performance in low- and middle-income countries.

**Methods:**

A review of peer-reviewed articles, reports, and global data collection tools was conducted to identify key measurement domains in monitoring CHW performance. Three consultations were successively convened with global stakeholders, community health implementers, advocates, measurement experts, and Ministry of Health representatives using a modified Delphi approach to build consensus on priority indicators. During this process, a structured, web-based survey was administered to identify the importance and value of specific measurement domains, sub-domains, and indicators determined through the literature reviews and initial stakeholder consultations. Indicators with more than 75% support from participants were further refined with expert qualitative input.

**Results:**

Twenty-one sub-domains for measurement were identified including measurement of incentives for CHWs, supervision and performance appraisal, data use, data reporting, service delivery, quality of services, CHW absenteeism and attrition, community use of services, experience of services, referral/counter-referral, credibility/trust, and programmatic costs. Forty-six indicators were agreed upon to measure the sub-domains. In the absence of complete population enumeration and digitized health information systems, the quality of metrics to monitor CHW programs is limited.

**Conclusions:**

Better data collection approaches at the community level are needed to strengthen management of CHW programs and community health systems. The proposed list of metrics balances exhaustive and pragmatic measurement of CHW performance within primary healthcare systems. Adoption of the proposed framework and associated indicators by CHW program implementors may improve programmatic effectiveness, strengthen their accountability to national community health systems, drive programmatic quality improvement, and plausibly improve the impact of these programs.

## Background

The landmark 1978 Declaration of Alma-Ata formally entrenched for the first time a political declaration that enshrined health as a human right within the global health agenda and emphasized primary health care (PHC) as a key mechanism for achieving health for all [1]. The 40th anniversary of the Declaration of Alma-Ata was a global call to re-invigorate efforts to achieve health for all through strengthening PHC systems [2, 3]. Mounting evidence since 1978 in low-and-middle-income countries (LMICs) has shown that focusing on provision of health services at the community level not only leads to more efficient and equitable use of health resources and better health outcomes [4], but also is a consistent component of strong, effective health systems. Since Alma-Ata, community health worker (CHW) programs in LMICs have been promoted to strengthen broad-based health efforts within community settings. CHWs, who for the purposes of this paper, refer to frontline health workers with up to 6 months of initial training, serve as the first point of contact for community members, especially for individuals living in low-income or rural communities whose access to facility-based health care may be limited. Often as community members themselves, CHWs possess a unique understanding of the local context, including barriers and facilitators to accessing timely and quality PHC, and can facilitate the most effective linkages to care.

Despite decades of interest and renewed commitment to expand Universal Health Coverage (UHC) by scaling up CHW programs, a universal, standardized system for empirically measuring the effectiveness of CHW programs does not yet exist [5–7]. Measurement is limited by the lack of an accepted and pragmatic set of theoretically grounded and validated indicators [8]. The integrated Community Case Management (iCCM) Framework was an attempt to provide guidance on measurement and metrics of iCCM programs; however, it has few metrics measured at the community level [9]. There is a need for consensus on measuring CHWs’ performance as a part of the larger community health system, broadly defined as “set of local actors, relationships, and processes engaged in producing, advocating for, and supporting health in communities and households outside of, but existing in relationship to, formal health structures” [10–13]. In practice, community health systems may include the enabling environment in which CHWs work, including household-level caregivers, other formal and informal healthcare providers, organizational intermediaries including non-governmental and faith-based organizations, other government sectors such as housing and education, among others [11]. As the link between individuals, communities, and health facilities, CHWs must be fluent in navigating the formal, facility-based health system, while maintaining their relationships within the community where they work [11, 14]. Consequently, the measurement of CHW performance needs to account for not only the activities of the CHW, but also those of the community health system that support the CHW. Experts have called for the recognition of the community health system as its own, unique sub-system of the health system, articulating a need to standardize the way performance and success of this sub-system is measured [11].

Addressing this gap in standardized metrics for assessing performance of community health systems is one of the main goals of Population Council’s Frontline Health (FLH) project, supported by the Bill & Melinda Gates Foundation and implemented in partnership with Last Mile Health. USAID, UNICEF, and the Bill & Melinda Gates Foundation are working together around a set of jointly defined investment priorities that advance frontline delivery of and community engagement in primary health care. The Integrating Community Health (ICH) collaboration focuses on catalytic partnerships for system strengthening; measurement, learning, evaluation, and accountability; and, advocacy and pathways to scale. FLH is a partnership of the Population Council and Last Mile Health with USAID, UNICEF, and ICH partners in Bangladesh, Democratic Republic of the Congo, Haiti, Kenya, Liberia, Mali, and Uganda.

This partnership builds on the momentum set forth by the Kampala Declaration and the Agenda for Global Action [15] for higher commitment by governments and development partners to strengthen health workforce, and supports the recently released WHO guideline on health policy and system support to optimize CHW programs through the generation of tools and best practices to improve the design, implementation, performance, and evaluation of CHW programs [16]. The Frontline Health project interacts with country and global stakeholders to contribute to the objective of advancing metrics and evidence for community health. As part of this effort, key learning and research priorities for countries considering greater institutionalization and professionalization of CHW programs have already been identified in another manuscript [17].

Here, we distill lessons from the literature and expert consultations to propose the Community Health Worker Performance Measurement Framework. The goal of this framework is to guide governments and implementing agencies in the development of priority standardized metrics for measuring the performance of CHW programs within the context of the broader system within which they operate. In this article, we describe the process of developing the framework and associated metrics, identify considerations for measurement of CHW program performance, and articulate future considerations for developing a robust agenda for monitoring community health systems at the country level.

## Methods

### Review of evidence

Community health literature from peer-reviewed databases and online knowledge management centers (CHW Central and Human Resources for Health (HRH) Global Resource Center), as well as publications from multilateral organizations, were reviewed to identify broad measurement domains in CHW program monitoring. A total of 85 reports from the CHW Central and 300 from the HRH Global Resource Center were screened for relevance. Peer-reviewed articles were identified using a convenience snowball sampling and included if they identified critical measurement gaps to improve CHW programs. This information was abstracted and consolidated to inform the measurement domains for the framework. At first, the review was aimed at identifying a framework in the existing literature that would comprehensively identify priority metrics for measuring CHW programs, instead of developing a new framework. We identified 34 frameworks (Box 1) that peripherally addressed this objective, including but not limited to the CHW Assessment and Improvement Matrix (CHW AIM) [18], CHW logic model proposed by Naimoli et al [6], USAID Community Health Framework [19], and the Primary Health Care Performance Initiative (PHCPI) conceptual framework [20]. While these frameworks were useful in identifying areas of measurement appropriate to community health, they were not explicitly developed for operationalizing measurement of CHW programs [6, 18–20]. Given this, a new framework was proposed that leverages these existing frameworks and is pragmatically geared towards monitoring community health worker programs.

**Fig. 1:**
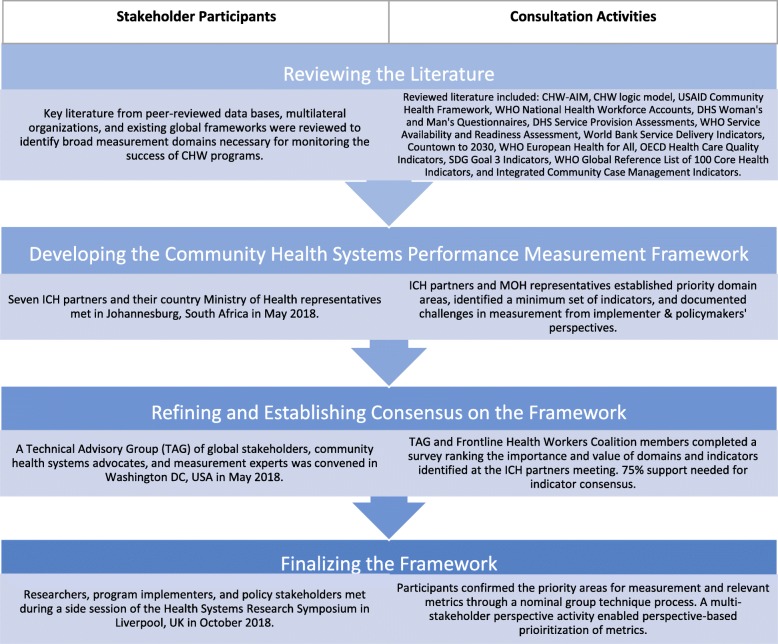
Development of the Community Health Worker Performance Measurement Framework

The review of literature and existing frameworks helped identify and define sub-domains of a draft Community Health Worker Performance Measurement Framework. This was refined further based on a series of consultations as described below (Fig. 1).

### Concept development

Four critical considerations guided the development of the framework:

1. Measurement of CHW performance should consider system-level processes including the role of governance, policy, internal and external investments, and other supportive mechanisms, and account for the broader community health system that influences CHW program performance.
2. In countries without centralized coordination, NGOs typically play an outsized role in implementation of CHW programs, with divergent CHW roles, responsibilities, and timelines. To account for this variation, we recommend the use of existing standardized metrics (e.g., for iCCM, HIV, TB, family planning, maternal health) to measure downstream impacts of services on a specific outcome area.
3. While several factors contribute to the success of CHW programs, the proposed indicators around the framework focus on aspects that are amenable to and critical for the purposes of measurement of CHW performance. For multidimensional and critical concepts such as community empowerment and job satisfaction, formative work is currently being undertaken by the Population Council within the Frontline Health project to develop scale-based metrics.
4. Balance short-term demand for data to inform programmatic activities with the long-term data needed to understand program effectiveness.

### Operationalizing measurement constructs as indicators

To operationalize the framework sub-domains, the monitoring, evaluation, and learning (MEL) plans of the seven ICH NGO partners were reviewed to identify pragmatic indicators across a range of contexts [21–27]. These indicators had been operationalized by the implementing NGOs, with varying levels of success across a wide range of contexts. The ICH NGO partners’ proposed indicators were mapped on the frameworks’ sub-domains. Where appropriate, the language was revised to generalize the indicators, and new measurement sub-domains were added or combined. Next, indicators in the peer-reviewed literature were identified from 383 reports in the Health Care Provider Performance Review database using the filters of “lay health worker” and processes of care outcomes [28]. A desk review of global data collection tools was conducted to identify existing global indicators that evaluate CHW performance. Reviewed sources are as follows: WHO National Health Workforce Accounts [29], Woman’s and Man’s Questionnaires from Demographic and Health Surveys (DHS) [30], DHS Service Provision Assessments (SPA) [31], WHO Service Availability and Readiness Assessment (SARA) [32], World Bank Service Delivery Indicators (SDI) [33], CHW Assessment and Improvement Matrix (CHW AIM) [18], Countdown to 2030 [34], WHO European Health for All [35], OECD Health Care Quality Indicators [36], SDG Goal 3 Indicators [37], WHO Global Reference List of 100 Core Health Indicators [38], and Integrated Community Case Management (iCCM) indicators [9]. Each of these sources was systematically searched for its inclusion of indicators that explicitly measured CHW performance at the community level.

### Consultations and prioritization of metrics

Three key consultations were held to align findings from the literature with expert opinion and stakeholder perspectives. The first two consultations followed a modified Delphi approach [39]. In May 2018, representatives of the seven ICH NGO partners and the Ministry of Health of each ICH country met in Johannesburg, South Africa. This group (*n* = 29) worked to prioritize measurement domain areas for the framework, clarify definition of each domain and identify a minimum set of feasible and efficient indicators as well as to document challenges in measurement from the perspective of implementers and policymakers [40]. Feedback solicited during the workshop was incorporated in a revised version of the framework. A Technical Advisory Group (TAG) of 25 distinct participants was then convened in Washington, DC, United States of America, to identify priority areas for measurement from the perspective of global stakeholders, community health system advocates, and measurement experts. During the TAG meeting, a structured web-based survey was administered to TAG members, as well as select members of the Frontline Health Workers Coalition who volunteered to participate. For each of the measurement sub-domains, the respondents were asked to determine its importance (response categories—yes, no, maybe). For each of the indicators under the sub-domains, respondents were asked whether the indicator was valuable (response categories—yes, no, unclear, unsure). Sub-domains that had greater than 75% support as important areas of measurement, were further discussed in plenary, and the associated indicators further refined. For sub-domains with less than 75% agreement on their level of importance, changes were made based on expert feedback alone. Results from the survey are presented in Additional file 1.

As a final step, in October 2018, a consultation was held in Liverpool, United Kingdom, during a side session of the Fifth Global Symposium on Health Systems Research. A meeting of a third set of 32 researchers, program implementers, and policy stakeholders, focused on community health systems, met to further validate the framework and indicators by confirming their priority areas for measurement and the relevant metrics through a nominal group technique process [41, 42]. Following this process, participants were asked in a group discussion format to adopt the lenses of (a) donors/international policymakers, (b) national/sub-national policymakers and managers, (c) program/service implementers, and (d) monitoring/evaluation specialists and researchers and prioritize metrics within the framework based on their perspective. Qualitative notes taken during this discussion, including priority areas and indicators documented during nominal group technique process and perspective-based presentations, were used to further clarify the indicators, refine the definitions, and contextualize them.

## Results

### Community Health Worker Performance Measurement Framework

**Fig. 2:**
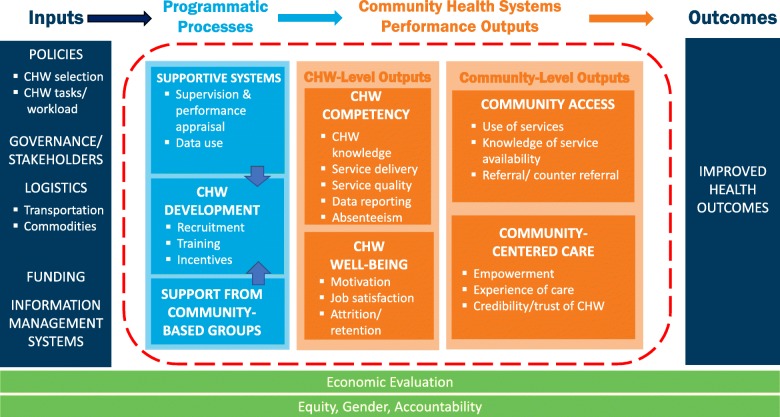
Community Health Worker Performance Measurement Framework

The Community Health Worker Performance Measurement Framework (Fig. 2), derived from iterative framework and indicator review and consultation, identifies critical areas for measuring the performance of CHW programs within their community health systems [43, 44]. While community health systems are inherently non-linear and complex, the framework structure uses the common input-process-output-outcome logic model approach and has four areas: *inputs*, programmatic *processes*, community health performance *outputs* (measured at the CHW level and at the community level), and *outcomes* [45]. Specific measurement domains and sub-domains are defined under each of these categories, with operational definitions in Table 1.

**Table 1:** Operational definitions of measurement constructs in the Community Health Worker Performance Measurement Framework

	Indicator Domain	Definitions/explanations	References
	**Inputs**		
1.	Policies	National-level policies that support the development and deployment of CHW programs	
	CHW selection	Age, education and other policy-supported requirements for being eligible to become a CHW are listed	[5, 14]
	CHW tasks/workload	Description of the role and tasks to be performed by a CHW from the community, CHW and health systems perspectives	[5, 18, 46, 47]
2.	Governance/stakeholders	Engagement with the Ministry, agencies and other stakeholders to support the CHW program	
3.	Logistics	Provisions, material and technological to support CHW functions.	
	Transportation	Provisions, either monetary (fare for busses) or physical (bicycle) for CHWs to physically access target population	[5]
	Commodities (including job aids)	Required equipment, medicines and supplies to deliver services, as well as resources such as job aids to support the quality of services	[5, 18, 46–48]
4.	Funding	Level of government/donor and other stakeholder investments in CHW programs in country	
5.	Information management systems	Support for CHW to document home visits including community-based health information systems, report visit-related data to the health system and link it to an assessment of CHW performance	[18]
	**Programmatic processes**		
	Indicator domains	Definition/explanation	References
1.	Supportive systems	Structural processes that influence CHW functions at various levels of the health system (facility/local/sub-national/national)	
A.	Supervision and performance appraisal	Consistent and continued support for problem solving, service delivery and skill development, including evaluation and supportive feedback on the work performed by the CHW in a set period	[10, 14, 18, 46, 47]
B.	Data use	The use of data by individuals at various levels of the health system to make decisions and improve operational processes	
2.	CHW development		
A.	Recruitment	How and from where a community health worker is identified, selected, and assigned to a community	[18]
B.	Training	Training is provided to the CHW to prepare for his/her role in service delivery and ensure s/he has the necessary skills to provide safe and quality care.	[5, 18, 46, 47]
C.	Incentives	Includes financial incentives such as salaries and bonuses, and non-financial incentives such as training, recognition, uniforms and other opportunities for advancement	[5, 6, 14, 18, 47]
3.	Support from community-based groups	Role that the community (any organization or group at the community level) plays in selection, supervision, offering incentives and providing feedback to the CHW	[5, 18, 46, 47]
	**Community health systems performance outputs—CHW level**		
	Indicator domain	Definition/explanation	References
4.	CHW competency	Degree to which CHW has the knowledge and skills necessary to carry out the assigned tasks	
A.	CHW knowledge	Degree to which CHWs have theoretical knowledge of counseling, preventative and curative and other tasks they are responsible for	[5, 6, 49]
B.	Service delivery	Quantity of promotional, preventive and curative services CHWs provide to community members	[6, 46]
C.	Service quality	Adherence to standards and procedures (counseling, health promotion, treatment accuracy)	[6]
D.	Data reporting	Regularity and completeness of CHW reports on the services they provide at the community level	[50]
E.	Absenteeism	Frequency with which CHWs do not carry out tasks	[6]
5.	CHW well-being	The overall well-being of the CHW may be seen as a measure of effectiveness of the system that supports the CHW program	
A.	Motivation	An individual’s degree of willingness to exert and maintain effort on assigned tasks; a CHW’s confidence, belief in his/her ability to produce a desired result	[5, 6]
B.	Job satisfaction	Degree to which CHWs derive personal satisfaction from serving the community and providing services	[5, 6]
C.	Attrition/retention	The rate at which practicing CHWs resign, retire or abandon their positions	[6, 14]
	**Community health systems performance outputs—community level**		
	Indicator domain	Definition/explanation	References
6.	Community access	Delivery of CH services in a timely manner within the client’s home/community OR clients’ physical/social access to CHW service	[5, 6, 49, 51]
A.	Use of services	Clients are routinely seeking and using promotional, preventive and curative services that CHWs offer	[5, 6]
B.	Knowledge of service availability	Clients’ ability to identify the location of CHWs and services they provide	[6]
C.	Referral/counter-referral	The acceptance and use of services provided at a health facility following referral by a CHW	[5, 49]
7.	Community-centered care	Community involvement, experience, and perceptions of services provided by CHWs	
A.	Empowerment	Both individuals and communities participate actively in community health activities	[49]
B.	Experience of care	Clients’ experience of services delivered by CHWs, including respectful care, and clients’ perception of quality of care.	[6]
C.	Economic evaluation	Comparison of two or more possible courses of action, with respect to the costs, consequences, and/or benefits of each	[52]
D.	Credibility/trust of CHW	Degree to which clients consider the services provided by CHWs to be credible and reliable	[6]

Bold entries are domain sections

The sections below detail measurement considerations, approaches for disaggregation, and data challenges for each of the programmatic process and performance output sub-domains under process and outputs in the framework, as well as recommendations for framing each measure from a gender and equity lens derived from the consultative and review process.

Table 2 identifies indicators for each of the measurement sub-domains and classifies them by their utility (high or moderate) for managers at the facility level, district level, and regional/national level, as well as suggests likely data sources. Inputs at the level of policy, governance, logistics, funding, and information management systems tend to have a large amount of variability in what might be appropriate to measure, given the country context, the maturity, the diversity of service delivery programs, and the degree of health system integration of the CHW program—therefore, specific indicators at this level should be reflective of what is most appropriate to understand the fidelity of program inputs. Given that CHW programs may focus on a wide range of context-appropriate health interventions, measurement of health outcomes attributable to the CHW program should also align with nationally or internationally recommended standardized coverage and impact health indicators.

**Table 2:** Illustrative indicators for community health worker performance measurement

Illustrative indicators		Indicator relevance/use by:			Suggested data sources**
		Facility manager/QI team	District managers	Regional and national MOH	
		H: High data use M: Medium data use			
**Domain 1: Supportive systems**					
	***Sub-domain A: Supervision and performance appraisal***				
1.	#/% of supervisors trained in management and supervision of CHWs	H	H	M	Training logs
2.	Ratio of CHWs to supervisors	H	H	M	HRIS
3.	#/% of supervisory visits that met the quality criterion	H	H	M	Special studies
4.	Average # of visits per supervisor to monitor/support CHW activities in the last month	H	H	M	Supervisor report/special studies
5.	#/% of CHWs who received a supervisory visit in the last 1–3 months that includes review of reports and data collected	H	H	M	CHW report
6.	Average # of supervisory contacts (in-person visits, phone calls, text messages, etc.) per CHW	H	H	M	CHW report
	***Sub-domain B: Data use***				
7.	#/% of health workers (CHWs/supervisors/health facility staff) who have access to client data AND who report using the data to make decisions about their provision of services	H	H	M	Special studies (CHW survey)
8.	#/% of national/sub-national/facility/community meetings in which data (from standardized reporting platforms etc.) are discussed/reviewed	H	H	H	Routine meeting minutes
9.	#/% of CHWs who have access to the client data they have collected (for follow-up) in the last 6 months	H	H	M	CHIS/HMIS
**Domain 2: CHW development**					
	***Sub-domain A: Recruitment***				
10.	#/% of CHWs who have been selected in alignment with selection criteria	M	H	H	HMIS/HRIS/training logs
11.	# of CHWs who have been selected/recruited	H	H	H	HRIS
12.	#/% of target communities/populations that have an assigned CHW	H	H	H	HRIS
	***Sub-domain B: Training***				
13.	#/% of CHWs who have received initial training	M	H	H	HRIS/training logs
14.	#/% of CHWs who have received follow-up training in the last 2 years	M	H	H	HRIS/training logs
15.	#/% of CHWs who have completed the certification program	M	H	H	HRIS/training logs
	***Sub-domain C: Incentives***				
16.	#/% of CHWs who have received their stipend in the last month	M	H	H	CHW report/supervisor report/special survey
17.	#/% of CHWs who have received a specific non-financial incentive	M	H	H	CHW report/supervisor report/special studies
**Domain 3: Support from community-based groups**					
18.	# of planning/review meetings held at the level of the local government to discuss CHW program performance	M	H	H	Meeting minutes/special studies
**Domain 4: CHW competency**					
	***Sub-domain A: CHW knowledge***				
19.	#/% of CHWs who have passed knowledge/competency tests (following training)	H	H	H	Training/accreditation logs
20.	#/% of CHWs who express that they feel confidence in their abilities to provide health education	H	H	H	Special studies
21.	#/% of CHWs who express confidence in their abilities to deliver basic healthcare services	H	H	H	Special studies
	***Sub-domain B: Service delivery***				
22.	Average # of home visits made by CHWs in the last month (indicator to be disaggregated by type of home visit—i.e., sick child visit, antenatal care)	H	H	M	CHW record/CHW report
	***Sub-domain C: Service quality***				
23.	#/% of CHWs who correctly identified the case/health problem (as per items in a checklist)	H	H	H	Special studies/supervisor report
24.	#/% of CHWs who correctly addressed (treated) the identified health problem (as per items in a checklist)	H	H	H	Special studies/supervisor report
25.	#/% of CHWs with all the key stock commodities in the last reporting period	H	H	H	CHW report
26.	Average time from onset of symptom to first contact with CHW	H	M	M	Special studies
	***Sub-domain D: Data reporting***				
27.	#/% of CHWs who submitted reports in the last month	H	H	M	CHIS/HMIS/supervisor report
28.	#/% of CHW reports submitted that were complete/did not have missing information	H	H	M	CHIS/HMIS/supervisor report
	***Sub-domain E: Absenteeism***				
29.	#/% of CHWs who reported on their activities in the last month	H	H	H	CHIS/HMIS
30.	# of days CHW has performed at least one CHW responsibility in the last month	H	H	H	Special studies/supervisor report
**Domain 5: CHW well-being**					
	***Sub-domain A: Motivation***				
31.	Composite metric	H	H	H	Special studies
	***Sub-domain B: Job satisfaction***				
32.	#/% of CHWs who expressed satisfaction with the community support they receive	H	H	M	Supervisor report/special studies
33.	#/% of CHWs who expressed satisfaction with the support they receive from health facility staff	H	H	M	Supervisor report/special studies
	***Sub-Domain C: Attrition/retention***				
34.	In the last 3 months, #/% of CHWs who have reported on their activities	H	H	H	CHIS/HMIS
**Domain 6: Community access**					
	***Sub-domain A: Use of services***				
35.	#/% of households who received at least one visit by a CHW in the last 3 months	H	H	M	Special studies
	***Sub-domain B: Knowledge of service availability***				
36.	#/% of community members that know the name of the community CHWs	H	H	M	Special studies
37.	#/% of community members who can name at least 3 services that the CHW provides	H	H	M	Special studies
	***Sub-domain C: Referral/counter-referral***				
38.	% of individuals referred by CHW to the health facility per 100 clients seen (and subset by reasons for referral)	H	H	H	CHIS/HMIS/CHW record
39.	#/% of clients that completed the referral at the health facility (referral completion)	H	H	H	CHIS/HMIS
40.	#/% of referred clients seen at receiving service (health facility) that is seen back at referring service (CHW) with complete counter-referral information (counter-referral)	H	H	H	CHIS/HMIS
41.	Average # of referrals made per CHW in the last month	H	H	H	CHW report/CHW record
**Domain 7: Community-centered care**					
	***Sub-domain A: Empowerment***				
42.	Composite metric	M	H	H	Special studies
	***Sub-domain B: Experience of care***				
43.	#/% of women/households who express satisfaction with services they received from the CHW in the last 3 months	H	H	H	Special studies/community scorecards
44.	#/% of women who report that in their interaction with the CHW they felt humiliated or disrespected (scale 1–5)	H	H	H	Special studies/community scorecards
	***Sub-domain D: Credibility/trust of CHW***				
45.	#/% of women/clients who report they trust the health information provided by the CHW	H	H	H	Special studies/community scorecard
46.	#/% of women/clients who report they trust the treatment services provided by the CHW	H	H	H	Special studies/community scorecard

Measurement Consideration 8: Equity, Gender, and Accountability: Each of the above indicators may be disaggregated by one or more of the following characteristics to assess possible equity gaps or disparities: education, ethnicity, family type, health risk-level, immigration/migrant status, language, marital status, occupation, refugee/asylee status, religion, sex, social capital, tribe, village size, wealth

Measurement Consideration 9: Economic Evaluation: While no extant routine or recommended indicators for measuring CHW program performance were identified, metrics for cost-related benefits of institutionalizing CHW programs and engaging communities are important to capture and require further exploration

**Special studies may include cross-sectional, intermittent surveys of a sample of CHWs, supervisors and/or community members. In case of quality of care, a special study might include direct observation of the CHW during a client interaction

*HRIS* Human Resource Information System: a national/sub-national HRH database or registry, either digital or paper-based (e.g., iHRIS), that manages health workforce information such as number of health workers (e.g., CHWs) recruited, trained, and on payroll

*CHIS/HMIS* Community Health Information Systems/Health Management Information Systems

CHW record: Routine records or community-based client registries maintained by CHW

CHW report: Summative reports on the number of home visits and types of client seen, typically submitted on a pre-determined schedule by CHWs to their supervisors

Supervisor report: Summative reports on activities of CHWs and other community-based logistics, routine submitted by supervisors to district or regional level administration

Bold entries are domain and sub-domain sections

Based on feedback from TAG members, the following areas had greater than 75% agreement as the most critical areas of measurement: attrition (100%), quality of services (95%), service delivery (95%), supervision (95%), experience of services (90%), use of services (90%), referral/counter-referral (85%), CHW absenteeism (80%), incentives (80%), performance appraisal (80%), credibility/trust (75%), data reporting (75%), data use (75%), and costs. While the measurement of costs, cost-benefit, and cost-effectiveness of CHW was unanimously considered vital, no specific indicators were recommended due to the variability in the functions of such programs. Measures around supervision and performance appraisal were combined due to the overlap in the associated metrics.

### Measurement considerations for core framework constructs

#### Supportive systems

A. Supervision and Performance Appraisal: Provision of routine support to CHWs is important for problem solving, skills development, motivation, and quality service delivery. An important existing gap is the frequent lack of contact with supervisors or former mentors at training institutions once the CHW graduates and is posted, especially in rural areas [53]. The measurement of the assignment and provision of supervisory visits is important and associated data might be routinely collected in training logs, CHW, and supervision reports. CHW performance appraisal and feedback from supervisors are key components of quality supervision and require assessment as well, possibly in a non-routine study. Alternately, digital and mobile job aids with algorithms and behavioral analytics could help operationalize quality assessments and provide remote supervision. The Perceived Supervision Scale (PSS) is a six-item scale that captures regular contact, two-way communication as well as joint-problem-solving, and has been validated in six countries [54]. We recommend the use and refinement of this measure across different contexts or, alternately, testing a modified PSS such that it captures elements of perceived value of CHW supervision, and the content and alignment of the supervisory visit with protocols. Alternatively, the quality of supervision may also be assessed through a spot check, where supervisors are accompanied by their managers on their supervisory visits, and feedback on the quality of supervision is provided after the visit based on observation.
B. Data use: The use of data by health workers at all levels of the health system can help with responsive feedback and quality improvement efforts. If data are being routinely collected and reported, it does not automatically imply that they are being used for decision-making. Assessment of data use may require special cross-sectional and ethnographic studies, to understand whether data are being used for decision-making and assess reasons for data use/disuse behaviors. Inclusion of this metric in the framework also highlights the need to have management structures in place such that those that are collecting the data, especially client data, also have access to that information [46].

#### CHW development

A. Recruitment: Several aspects of measurement of CHW recruitment are important, including understanding how many CHWs are recruited, their density per geographic area (e.g., by district, region), and disaggregating recruitment by the representation of CHWs from different types of communities (e.g., diverse ethnicities, socioeconomic backgrounds, and originating from that community, among others). At the national and sub-national levels, the number of CHWs that have been recruited need to be monitored for fiscal planning and ensuring adequate coverage. From a measurement perspective, it is important to assess the alignment between national CHW selection policies and the actual recruitment and to recognize information systems’ capacity to capture workforce turnover data. In some cases, education, language, or other requirements may limit recruitment of CHWs from marginalized communities [47]. In addition to the actual number of CHWs who have been recruited, it is also important to understand coverage, and quality and transparency of the recruitment process. Community participation in the recruitment process can facilitate community ownership and validation of CHWs within the communities where they work.
B. Training: Initial and ongoing knowledge and skills-based training is critical to prepare CHWs for their role in service delivery. Records on the numbers of CHWs trained are typically maintained at the program, or district level at which training occurs, and might be further aggregated at a sub-national level to assess alignment with national targets. Maintaining training records at the program or district level can be a challenge as these trainings are often led by external NGOs who may be equally challenged by information systems limitations to feasibly transfer or maintain training data. Identifying mechanisms to consolidate the number of CHWs trained and maintaining rosters of the topic areas on which training has been received is important to scaling programs and may require additional district-level oversight of data management. If a national certification program exists for CHWs, tracking the number of existing and new CHWs who have completed certification is important. Finally, ensuring that measurement of training comprises not just the technical content, but also the procedures around data entry and reporting is crucial. While the number of trainings held is somewhat routinely tracked, the actual competencies of CHWs are more complex to assess. Trainings may capture pre- and post-test knowledge scores, but efforts to assess the extent to which CHWs effectively apply their newly acquired skills are less frequently undertaken [50].
C. Incentives: While measurement of financial and non-financial incentives provided to CHWs is important, it is also a difficult area to routinely monitor. Where CHWs receive a pre-determined stipend, salary, or performance-based incentives, the receipt of these stipends might be routinely monitored through monthly/quarterly CHW or supervisor reports. Often, if CHW stipends are provided through supervisors or other personnel in the healthcare system, having routine measurement of whether the stipend was received can aid in curbing corrupt practices or mismanagement of funds. Non-financial incentives may come in many forms—they may be institutionalized by governments in the form of educational or promotional opportunities, or by communities in the form of social recognition of the CHW [17, 53, 55]. If an institutionalized non-financial incentive exists, it might be feasible to routinely measure it. If not, programs may consider identifying and disaggregating non-financial incentives that exist at the community level and assess them cross-sectionally.

#### Support from community-based groups

A. CHWs operate within the complex interplay of community and health systems. Engagement with and support from the communities in which they operate is critical to optimal functioning of CHWs, as also highlighted by the U.S. Government Evidence Summit [5, 16, 51, 56, 57]. It is important for communities to engage through a feedback mechanism, such as a community scorecard, with which they can assess challenges the community faces that can be used to provide feedback and targeted support to CHWs. Community-based groups such as village health committees, facility management committees, or other contextually appropriate local governance structures, including non-health sector groups, can play a role in identifying solutions to challenges. These community mechanisms are complex to measure routinely or in a binary format, and may be best measured through cross-sectional, intermittent, qualitative assessments.

#### CHW competency

A. Knowledge on specific technical subject matter underpins quality of service provision. Measurement of CHW knowledge may be considered at the end of each training cycle, or cross-sectionally, where new knowledge is being imparted and skills developed. In lieu of metrics to capture CHW knowledge, assessment of the actual performance of the CHW is more important, as knowledge does not always translate to practice, known as the “know-do” gap [58]. If the performance of CHWs does not meet required standards, an assessment of knowledge might be relevant if it is suspected that poor knowledge is the cause of poor performance.
B. Service delivery: The delivery of health services refers to the quantity of promotional, preventive, curative, and rehabilitative services CHWs provide to community members. Indicators under this domain pertain to the activity of the CHW, measured at the level of the individual CHW. CHW activities might be consolidated from CHW service delivery registers and routine reports. Indicators to assess CHW service delivery should be tailored to the individual CHW program context and the types of services provided by the CHW. The illustrative indicators in Table 2 may be further broken down by type of services for which the home visit was made. Note that measures of service coverage at the community level typically involve population-based surveys and are listed under “use of services”.
C. Service quality: Measurement of the quality of services provided by the CHW is perhaps one of the most critical measures to assess effectiveness of a CHW program, as historically, these programs have lost financial support when service quality does not meet required standards or varies substantially [59]. Service quality should be measured both from the technical or clinical perspective and the client’s perspective (captured under “experience of care”). Unless routine quality assessment and control measures are built into supervisory activities, the measurement of service quality may require non-routine studies. When measured routinely, assessment of quality may be done through spot checks by supervisors to ascertain quality of service delivery using a checklist, or by examining client ledgers or registers. Simple checklists implemented by supervisors to observe and assess existing quality of services or community follow-up tools utilized by community members themselves may provide immediate and comprehensive feedback to the CHW.
  If conducted as a non-routine study, assessment of quality at the community level poses logistical challenges that are not typically experienced when assessing quality at the facility level, where direct observation of client-provider interaction and exit interviews are the normative methods for quality assessment. CHW services vary widely by content area including routine counseling, linkages to health facilities, provision of certain interventions, and early detection and identification of health problems. Measures of CHW service quality should be tailored to specific CHW programs developed around a menu of potential service delivery priority areas. One indicator that is a common measure of quality is the timeliness of service receipt (from the first onset of symptoms).
D. Data reporting: Refers to the regularity and completeness, including data validity and verification, of reporting on the services CHWs provide at the community or household level [60]. The level of data reporting can skew our understanding of all other progress measures—plausibly CHWs who are more active in their communities are also reporting more regularly and accurately. Differences in types of reporting mechanisms could lead to data that is not easily comparable across settings.
E. Absenteeism: Refers to the frequency with which CHWs are absent from their routine responsibilities. In settings where CHWs are volunteers or part-time employees, a regular schedule of activities may not exist [61]. This makes assessment of absenteeism challenging. If CHWs are reporting on their activities with some level of regularity, that might be a proxy measure for their regularity in their roles as CHWs. In cases where CHWs are expected to collect and report digitally, such data could be routinely made available on data dashboards.

#### CHW well-being

A. Motivation: Refers to intrinsic and extrinsic factors that influence CHWs’ interest in and willingness to perform their jobs. The challenge of measuring motivation through specific indicators stems from its latency and multidimensionality as a construct and demands a scale-based approach. Motivation dimensions previously measured in community and primary health care settings include inertia towards one’s job; external financial rewards; self-worth contingencies including recognition from communities, supervisors, and colleagues; accepting the value of the job; internalizing the job’s value into one’s sense of self; and performing an activity for its own sake [62–64]. Motivation is best ascertained by CHW self-report through special surveys. Further investigation of adapted scales can potentially lead to the development of proxy metrics that could be included in routine community-integrated monitoring mechanisms.
B. Job satisfaction: Refers to a latent construct that captures a CHW’s sense of his or her ability to perform his or her job in a particular work climate [65]. Similar to motivation, job satisfaction is best suited to measurement using a scale-based approach that aggregates the attributes of a CHW’s job, including the job design and shared/participatory decision-making, empowerment, timeliness of decision implementation, availability of material supportive aides, support by communities in which they work, adequate feedback, recognition, management by supervisors, and mutual trust and cooperation among peers. While scale development approaches allow for refining measurement of CHW job satisfaction, and it is recognizably best measured through adapted scales in special surveys, two indicators that emerged as potential proxy metrics are presented in Table 2.
C. Attrition/retention: In comparison to absenteeism, measurement of attrition aims to capture the proportion of practicing CHWs who resign, retire, or abandon their positions. Given that CHWs may be volunteers, and their “employment” may often not be captured in official rosters, prolonged inactivity may be considered a proxy for attrition, where “prolonged” is a standard length of time determined at the country level, e.g., 1 month, 3 months [47]. The indicators presented in Table 2 are limited by the fact that some CHWs may not report regularly but may continue delivering services and/or intend to continue in their roles. As such, CHWs identified through monitoring this metric should be followed up in person.

#### Community access

A. Use of services: Community-level use of services is typically measured through population-based surveys. To the extent possible, these indicators should be aligned with extant national surveys that already capture indicators specific to CHW services. For example, the DHS measures the percentage of pregnant/recently delivered women who were visited by a CHW in the last 12 months, as well as several other health-area-specific indicators focused on use of services offered by CHWs. Table 3 presents a list of these indicators based on extant surveys. To ensure that programmatic CHW indicators are comparable with globally accepted measures, attention should be paid to alignment with these extant metrics.
B. Knowledge of service availability: Understanding the degree to which the community is aware of the presence and availability of a local health worker and the services they provide is critical to respond to community needs and priorities [5, 66]. Measuring this may require a population-based study.
C. Referral/counter-referral: Timely and appropriate referral from the community to the health facility is often considered one of the key functions of CHWs. Counter-referral from the health facility back to the CHW facilitates continuity of care in the home context of the client. One of the challenges with measuring referral is identifying the appropriate denominator (i.e., no. of clients eligible for referral per protocol). Such an assessment is not feasible to undertake routinely; therefore, referral may be measured per 100 or “X” clients seen by the CHW, disaggregated by reason for referral and used for comparisons across settings or over time [67, 68]. Measurement of counter-referral may only be pertinent in cases where continuity of care is recommended, as in the care of several chronic diseases. For example, if a child was referred to the facility for treatment of diarrhea, unless it is severe, there may not be a reason for counter-referral. However, if a client is referred to the health facility for suspected hypertension, counter-referral may be more appropriate. Measurement of counter-referral may also not be feasible in the absence of advanced information technology and digital systems that link facility records back to the community.

**Table 3:** Standard CHW service delivery metrics that are currently measured at the community level in national surveys

Indicator	Source
% women seen by CHW at first check after most recent delivery (following facility-based delivery)	DHS 7 Woman’s Questionnaire
% of women visited by a CHW in the last 12 months	DHS 7 Woman’s Questionnaire
% of women who reported talking with a field worker about family planning in the last 12 months	DHS 7 Woman’s Questionnaire
% of women who saw a CHW for antenatal care services for most recent pregnancy	DHS 7 Woman’s Questionnaire
% babies seen by CHW at first check after most recent delivery (Following facility-based delivery)	DHS 7 Woman’s Questionnaire
% women who were seen for PNC services by a CHW after leaving the health facility	DHS 7 Woman’s Questionnaire
% of babies seen by CHW within first 2 months of life	DHS 7 Woman’s Questionnaire
% of children under 5 seen by a CHW for diarrhea	DHS 7 Woman’s Questionnaire
% of children under 5 seen by a CHW for fever	DHS 7 Woman’s Questionnaire
% of women who obtained condoms from CHWs at time of last intercourse	DHS 7 Woman’s Questionnaire
% of men who discussed family planning with CHW	DHS 7 Man’s Questionnaire
% of men who obtained condoms from CHWs at time of last intercourse	DHS 7 Man’s Questionnaire
% of circumcised men who were circumcised by a CHW	DHS 7 Man’s Questionnaire
% of mothers who received postnatal care within 2 days of childbirth (regardless of place of delivery)	Countdown to 2030
% of mothers and babies who received postpartum care within 2 days of childbirth (regardless of place of delivery)	WHO Core 100 (2015)
# of health workers per 1 000 population (physicians, nurses and midwives, community health workers, etc.)	WHO IPCHS Global
% of mothers and babies who received postpartum care within 2 weeks/2 days of childbirth (regardless of place of delivery)	WHO IPCHS Global
# of CHWs trained and deployed for iCCM per 1 000 children under five in target areas	Integrated Community Case Management
Ratio of CHWs deployed for iCCM to iCCM supervisors	Integrated Community Case Management
Proportion of CHWs who received at least one administrative supervisory contact in the prior 3 months during which registers and/or reports were reviewed	Integrated Community Case Management
Proportion of CHWs who received at least one supervisory contact during the prior 3 months during which a sick child visit or scenario was assessed, and coaching was provided	Integrated Community Case Management
Proportion of CHWs who demonstrate correct knowledge of management of sick child case scenarios	Integrated Community Case Management
Proportion of sick children visiting a trained CHW who receive correct case management from that CHW	Integrated Community Case Management
Proportion of CHWs trained in iCCM who are providing iCCM 1 year after initial training	Integrated Community Case Management
Proportion of sick children who were taken to an appropriate provider (appropriate provider and aspects of timeliness defined by country protocols) (reported separately for each iCCM condition)	Integrated Community Case Management
Proportion of children recommended for referral who are received at the referral facility	Integrated Community Case Management
Proportion of CHWs targeted for iCCM who are trained and providing iCCM according to the national plan	Integrated Community Case Management
Proportion of CHWs (or iCCM sites in cases of multiple CHWs/area) treating at least X cases per month (to be defined locally)	Integrated Community Case Management
Proportion of overall treatment coverage of diarrhea and malaria being provided through iCCM by CHWs (reported separately for each iCCM condition)	Integrated Community Case Management
Proportion of CHWs targeted for iCCM who are trained and providing iCCM according to the national plan	Integrated Community Case Management
Proportion of CHWs who correctly count respiratory rate	Integrated Community Case Management
Proportion of sick children provided first dose of treatment in the presence of a CHW	Integrated Community Case Management
Proportion of sick child cases recommended for referral by the CHW	Integrated Community Case Management
Proportion of sick children under five in iCCM target areas taken to iCCM-trained CHWs as first source of care	Integrated Community Case Management
Number and proportion of cases followed up according to country protocol after receiving treatment from CHW	Integrated Community Case Management
Proportion of caregivers in target areas who know of the presence and role of their CHW	Integrated Community Case Management
Proportion of CHWs whose registers show completeness and consistency between classification and treatment	Integrated Community Case Management

#### Community-centered care

A. Empowerment: Community members’ agency—awareness of and access to—community health services as well as the participation or engagement of communities in shaping and maintaining community health services (including CHW programs) is a critical potential outcome of CHW programs. Given the multidimensional and highly contextual nature of empowerment, it is a difficult concept to capture through a standardized metric [49, 69]. Though proxies such as numbers of community meetings held, numbers of community members involved in CHW and facility feedback, and community health system-level contributions emerged in our discussions, these fall short given the varied definitions of empowerment and community-integrated governance structures across countries. Rather, cross-sectional special surveys alongside qualitative methods that describe the process, content, and relational dynamics (e.g., of community meetings) are required to assess empowerment. Adapting scales of individual, organizational, and community empowerment that have been developed in the context of health promotion can serve as a starting point to develop composite measures of empowerment as related to specific community health system components [70].
B. Experience of care: Understanding and measuring the experience of care from the client’s perspective is critical, but complex to standardize in the context of community health systems. This construct often overlaps with service use and technical service quality for a particular health area, though offers the subjective understanding of the interaction between client’s interactions with a CHW. Table 2 recommends that experience of care is measured through two indicators: levels of satisfaction with services received by a CHW in the last 3 months and the client perception of respectful care. Experience of care can be captured through self-report and, to some extent, observation by other community members or a trained data collector, close in timeframe to the service delivery (e.g., within 3 months) to avoid recall bias [71]. Composite measures to capture aspects of experience of care should be developed, validated, and incorporated into supervisory visits.
C. Credibility/trust of CHW: The degree to which clients consider the CHWs, and ultimately the services provided by CHWs, to be credible and reliable impacts the use of CHW services. While client trust in health information and services provided by the CHW are presented as proxy metrics in Table 2, these are not validated nor reflect the multidimensionality of trust within CHW-client relationships (includes dimensions of honesty, confidentiality, competency, and mutual respect, and partnership in health care decision-making processes) [72]. Quantitative measures of trust in CHWs should be adapted from PHC facility settings, contextualized to health areas and socio-cultural contexts through consensus-building methods, and evaluated through special surveys and scale-based approaches that are best suited to capturing latent constructs like trust [72]. Based on these studies, more realistic and relevant proxies can be investigated and recommended.

#### Measurement considerations for equity, gender, and accountability (see Table 2)

Equity, gender, and accountability are critical aspects to consider across most measurement domains [66, 73, 74] that require various qualitative and quantitative measurement methods from an array of stakeholder perspectives. Two primary concepts around equity of community health systems warrant discussion—first, the equitable selection and access to training and growth opportunities for CHWs themselves; second, the activities and inherent biases of the CHW and the health system differentially affect use and quality of services received by different community groups. When feasible, metrics should be disaggregated by place of residence, socioeconomic status—including education and wealth, sex, age distribution, occupation, social capital, language, religion, ethnicity, tribe, family type, health risk-level, village-size, marital status, immigration/migrant status, and refugee/asylee status (Table 2 footnotes). Disaggregation by equity-promoting variables requires a substantial commitment to advancing equity and utilizing accompanying resources. As a starting point, three levels of disaggregation, in alignment with the DHS might be considered—by wealth quintile, by level of education, and by place of residence (i.e., urban and rural) [75]. For allocation of resources at a national or sub-national level to the districts, performance metrics should be available, disaggregated by district to facilitate appropriate allocation of resources. We note here that equity should not be misunderstood for equality—while equality deals with fairness through equal distribution of resources, equity is concerned with need-based resource allocation even if that means unequal distribution [76].

Gender (in) equity may broadly refer to gender-related barriers to CHW performance, any unique needs and protections expressed by CHWs as a result of their gender (e.g., female CHWs may have certain safety concerns in some locations), minority identity in a particular context, and power relations facing CHWs within the health system hierarchy or community governance structures [74, 77]. These power relations relate to the attitudes around and interactions between CHWs, their supervisors, and other health workers or community workers. Measurement considerations might entail understanding the gender, ethnic, or age profiles of the cadre of CHWs and how that might affect perceptions of safety, interaction with the community and other health personnel in the health system, and overall motivation to continue working as a CHW. Beyond the need for capturing disaggregated metrics and needs of CHWs, who occupy lower positions in the health system hierarchy, it is important to consider policy-relevant metrics that support female health workers in particular, such as equitable hiring practices, uncompensated leave, sexual harassment, and discriminatory training policies [78]. Given that CHWs are typically responsible for all members of the community and given the expanding scope of their responsibilities (i.e., beyond traditional RMNCH care to include aging and non-communicable disease management), gender balance of recipients of care from the CHW at the community level is also important to consider.

Accountability in the context of community health systems is multifold, challenging to measure, but highly relevant in understanding how to institutionalize CHW programs and community health systems as agreed on by the TAG and literature [66]. It refers to the dual accountability of the CHW to the community for timely delivery of services and to the health system to perform required tasks, and to the responsiveness of the health system and communities to CHWs, namely by providing a supportive work environment, remuneration for service, and systematic feedback. Two perspectives might be considered around the measurement of accountability: as an outcome, which could be captured by policy-level inputs (e.g., funding integration in national and sub-national budgets and protective regulation around CHW scope) and local administrative government status reports, and as a governance and health system process that includes community participation in planning and review of CHW activities. Across the framework, indicators capture the types of support, remuneration (incentives), and recognition/systematic feedback that a CHW may receive from the government, health systems, and community, as well as the benefits the community receives from the activities of the CHW.

#### Economic evaluation

Emerging health system needs involve studies on the cost of scale up, particularly returns on investment in community health systems; economic evaluation metrics specific to community health systems better enable justification of health investments at the national level. However, no extant routine or recommended indicators around economic evaluation of CHW programs were identified [50]. Economic-cost-related metrics pose a challenge and require consideration of deaths or complications averted by CHW programs and comparative out-of-pocket cost savings models with respect to preventative care seeking from CHW and facilities. Ideally, economic evaluation metrics would involve various perspectives—health systems, societal, etc.—to reflect savings for households, facilities, or administrative/governments [17, 79].

## Discussion

The recommended domains (Fig. 2) and indicators (Table 2) are the result of extensive input from community health systems experts and practitioners in the field. The experts ranged from academics to officials from ministries of health across seven countries. A final consultation with practitioners in Liverpool, United Kingdom, validated the results; in Liverpool, participants debated the strength and value of indicators but did not identify new, actionable metrics. The experts also agreed that these recommendations strike a balance between a pragmatic minimum set of indicators and a more exhaustive set of metrics that would be costly and impractical to measure with regularity. The proposed list of metrics is not comprehensive; rather, it is presented as a point of reference to assist in standardizing metrics for CHWs.

CHWs work in a wide variety of contexts: from institutionalized, salaried roles well-integrated in the public health sector [13], to informal, volunteer-based work acting as important community educators and linkages between communities and the health system. CHWs may also deliver multiple interrelated and interdependent interventions simultaneously. Their services may involve activities across different levels of the health system, including referrals made to the health facilities [54]. This variability in their roles and range of services contributes to the challenge of standardizing metrics to measure the performance of CHW programs and attribute their impact on health outcomes [48].

The appropriate selection of indicators depends on the maturity of the community health system, as well as the types of data that are considered most critical to advocate for such community-based interventions nationally. The maturity of a CHW program can be observed in the degree to which the community health system is integrated with the formal health system (at national, regional, district, and facility levels). In some contexts, CHW programs operate as disparate stand-alone, NGO or private sector-led programs that may run in parallel to the public health system. In more mature settings, CHW programs are formally aligned with well-established government policy, with a formal governance structure, funding support, training agenda, job description, appropriate support from public health facilities, and efforts to integrate care from the community to the health facility. Within the proposed framework, some performance indicators will be more relevant to early CHW initiatives, while others will only become useful as the CHW service delivery layer becomes fully integrated into the health system.

The framework in Fig. 2 articulates measurement domains that should be captured by CHW programs, and the illustrative indicators in Table 2 represent a more pragmatic approach reflective of the limitations in standardized indicators, capacity for data collection and aggregation at the community level, and limited availability of community-based census or “denominator” data. The framework is limited to CHW programs targeted at health outcomes. However, the authors recognize the need for a multisectoral approach at the community level and the potential contributions of CHWs in education, agriculture, and other sectors. Some sub-domains have missing illustrative indicators (e.g., economic evaluation); others have indicators (e.g., referral/counter-referral) where methods of collection are challenging and under-developed resulting in a large amount of variability in the feasibility of measurement; still others have composite metrics (e.g., community empowerment) that are not validated or are multidimensional and highly contextual. Indicators for inputs (existing governance, policy, funding, and information systems) were not included largely due to their high variability across CHW program contexts. The framework and approach does not provide country-specific guidance for actionable use of particular indicators. Country-level adaptation and testing of indicators in practice can overcome this limitation.

These limitations highlight opportunities to strengthen the measurement of community health systems. To facilitate standardization, development and validation of comprehensive indicators is important. Advances on this front are ongoing, as in the case of the recently proposed six-item perceived supervision scale that has been validated in seven languages [54]. In practice, a more limited subset of the proposed metrics that have been validated and found most valuable to global and national decision-making should be identified and disseminated. An example of metrics prioritization is seen in Liberia, where a focus on quality has led to continued optimization of a nationally implemented CHW program. It should be noted that in emergency and fragile settings, CHW programs often emerge organically from the needs of the community. The most pertinent areas of measurement for such settings need to be identified and tested. While we engaged a number of national and global stakeholders in the development of this framework, its ongoing development would benefit from wider perspectives. The framework does not cover the full range of the community health system space, which involves more multisectoral players.

Of critical importance is the need to strengthen community health information systems (CHIS). Where previously, much of the community-level data collection and aggregation occurred manually on paper, the rapid digitization of health service data and data collection using mobile devices has opened new frontiers for enhancing CHIS performance [60]. In advancing primary health care, the 2018 Astana Declaration emphasizes the importance of building systems to collect “disaggregated, high quality data to improve information continuity, disease surveillance, transparency, accountability and monitoring health system performance”, and emphasizes investments into appropriate technology to facilitate this [51]. Several parallel investments are already ongoing. For example, the Health Data Collaborative community data sub-group aims to harmonize and endorse standards for CHIS to maximize integration with national health information systems. At the global level, the National Health Workforce Accounts were proposed at the 69th World Health Assembly and present a set of 78 indicators that can be collected nationally to improve the availability, quality, and use of data on human resources for health [29]. The Primary Health Care Performance Initiative supports countries to measure the most critical indicators to advance primary care [52]. As each of these tools and resources mature, intersections across them need to be explored. For example, the proposed framework and indicators herein can potentially support the assessment of the 10 programmatic components proposed by the most recent version of the CHW AIM Program Functionality Matrix [80]. Given the recent reinvigoration of investments in primary health care and community health systems, coordination across different initiatives to leverage existing work and avoid duplication will also be prudent. Efforts to support the standardization and collection of data must be accompanied by training and support to develop numeracy and skills to use data for decision-making.

## Conclusions

The proposed framework and indicators are a critical first step to addressing a long-acknowledged gap in identifying relevant, pragmatic, and contextually appropriate indicators to monitor the performance of CHW programs. Indicators are presented with practical insights and recommendations for routine and special study methods for data collection as well as reflections on integrating CHW performance indicators into routine health information systems. Adoption of the proposed indicators can guide the development of a robust monitoring system for CHW programs, help improve day-to-day programmatic performance, and in the long run have an impact on improved health outcomes. However, the authors emphasize that systems and resources to capture and utilize data at the community level face practical challenges far greater than those experienced in data capture at the level of the facility. We present this framework and indicators to generate a conversation and iteratively develop stronger systems to monitor CHW programs.

## Supplementary information

**Additional file 1.** TAG Survey for Metrics Development: May 2018, 25 respondents.

## Data Availability

Data sharing is not applicable to this article as no datasets were generated or analyzed during the current study.

## Abbreviations

CHIS: Community health information system
CHW: Community health worker
CHW AIM: Community Health Worker Assessment and Improvement Matrix
DHS: Demographic and Health Survey
FLH: Frontline Health Project
iCCM: integrated Community Case Management
ICH: Integrating Community Health
MEL: Monitoring, evaluation, and learning
PHC: Primary health care
PHCPI: Primary Health Care Performance Initiative
PSS: Perceived Supervision Scale
SARA: Service Availability and Readiness Assessment
SDI: Service Delivery Indicators
SPA: Service Provision Assessment
TAG: Technical Advisory Group
USAID: United States Agency for International Development

## References

[1] International conference on primary health care - Alma Ata, USSR 6-12 September 1978. Declaration of Alma-Ata 1978. 43969

[2] Chapman A (2018). ALMA-ATA at 40: revisiting the declaration.

[3] Schuftan C (2018). ALMA-ATA at 40: primary health care remains key to health for all—now.

[4] Starfield B, Shi L, Macinko J (2005). Contribution of primary care to health systems and health. Milbank Q..

[5] Kok MC, Dieleman M, Taegtmeyer M, Broerse JEW, Kane SS, Ormel H (2015). Which intervention design factors influence performance of community health workers in low- and middle-income countries? A systematic review. Health Policy Plan.

[6] Naimoli JF, Frymus DE, Wuliji T, Franco LM, Newsome MH (2014). A community health worker “logic model”: towards a theory of enhanced performance in low- and middle-income countries. Hum Resour Health.

[7] Rowe AK, De Savigny D, Lanata CF, Victora CG (2005). How can we achieve and maintain high-quality performance of health workers in low-resource settings?. Lancet..

[8] Perry HB, Zulliger R, Rogers MM (2014). Community health workers in low-, middle-, and high-income countries: an overview of their history, recent evolution, and current effectiveness. Annu Rev Public Heal.

[9] Indicator Guide: Monitoring and evaluating integrated community case management. 2013. 10.1093/heapol/czv129 PMC4916319 26758538

[10] Kok MC, Broerse JEW, Theobald S, Ormel H, Dieleman M, Taegtmeyer M (2017). Performance of community health workers: situating their intermediary position within complex adaptive health systems. Hum Resour Health.

[11] Schneider H, Lehmann U (2016). From community health workers to community health systems: time to widen the horizon?. Heal Syst Reform.

[12] USAID, UNICEF. Institutionalizing community health conference 2017. https://ichc2017.mcsprogram.org/.

[13] Pfaffmann Zambruni J, Rasanathan K, Hipgrave D, Miller NP, Momanyi M, Pearson L (2017). Community health systems: allowing community health workers to emerge from the shadows. Lancet Glob Heal..

[14] Campbell C, Scott K (2011). Retreat from Alma Ata? The WHO’s report on task shifting to community health workers for AIDS care in poor countries. Glob Public Health..

[15] World Health Organization, Global Health Workforce Alliance. The Kampala Declaration and a genda for global action. Geneva; 2008.

[16] World Health Organization. WHO guideline on health policy and system support to optimize community health worker programmes. Geneva; 2018. 30431747

[17] Agarwal S, Kirk K, Sripad P, Bellows B, Abuya T, Warren C (2019). Setting the global research agenda for community health systems: literature and consultative review. Hum Resour Health.

[18] Crigler L, Hill K, Furth R, Bjerregaard D (2011). Community health worker assessment and improvement matrix (CHW AIM): a toolkit for improving community health worker programs and services.

[19] USAID, Dalberg Global Development Advisors. Community Health Framework: distilling decades of agency experience to drive 2030 Global Goals. USAID, Advisers DGD, editors.

[20] Veillard J, Cowling K, Bitton A, Ratcliffe H, Kimball M, Barkley S (2017). Better measurement for performance improvement in low- and middle-income countries: the primary health care performance initiative (PHCPI) experience of conceptual framework development and Indicator selection. Milbank Q.

[21] Aga Khan Foundation M (2017). Strengthening the “Soins essentiels dans la communauté” Strategy Project Monitoring Evaluation and Learning Plan.

[22] Zanmi Lasante (2017). Scaling up agents de Santé communautaire polyvalent in Haiti monitoring Evaluation and learning plan.

[23] Save the Children (2017). Improving community health workers program performances through harmonization and community engagement to sustain effective coverage at scale in Bangladesh Monitoring Evaluation and Learning Plan.

[24] Humana People to People Congo (2017). Strengthening the CHW Systems in Urban and Rural Congo Monitoring Evaluation and Learning Plan.

[25] LVCT Health Kenya (2017). Sustaining quality approaches for locally embedded community health services (SQALE) Monitoring Evaluation and Learning Plan.

[26] Pathfinder International (2017). Integrated systems strengthening for CHW programming (Uganda) monitoring Evaluation and learning plan.

[27] Last Mile Health (2017). CHWs for ALL (Liberia) Monitoring Evaluation and Learning Plan.

[28] Rowe AK, Rowe SY, Peters DH, Holloway KA, Chalker J, Ross-Degnan D (2018). Effectiveness of strategies to improve health-care provider practices in low-income and middle-income countries: a systematic review. Lancet Glob Heal.

[29] World Health Organization. National Health Workforce Accounts: A Handbook. Geneva; 2017.

[30] Bangladesh Demographic and Health Survey 2014 (2016). Dhaka, Bangladesh: NIPORT, Mitra and Associates, and ICF International.

[31] MEASURE DHS (2012). Service Provision Assessment Survey: Inventory Questionnaire.

[32] World Health Organization. Service Availability and Readiness Assessment: An annual monitoring system for service delivery Version 2.2. Geneva; 2015.

[33] The World Bank (2017). Service Delivery Indicators.

[34] Countdown to 2030, WHO, UNICEF (2016). Countdown to 2030: Maternal & Newborn, Child and Adolescent Health Indicators.

[35] WHO Regional Office for Europe (2018). European Health for All database.

[36] OCED.Stat (2019). Health Care Quality Indicators.

[37] Inter-Agency and Expert Group on SDG Indicators (2016). SDG 3: Targets & Indicators.

[38] World Health Organization (2018). 2018 Global Reference List of 100 Core Health Indicators (plus health-related SDGs).

[39] Hsu C-C, Sandford BA (2007). The Delphi technique: making sense of consensus. Pract Assessment Res Eval.

[40] Summary report integrating community health partners’ metrics workshop. Washington D.C.: Population Council; 2018.

[41] Foth T, Efstathiou N, Vanderspank-Wright B, Ufholz L-A, Dutthorn N, Zimansky M (2016). The use of Delphi and nominal group technique in nursing education: a review. Int J Nurs Stud.

[42] McMillan SS, King M, Tully MP (2016). How to use the nominal group and Delphi techniques. Int J Clin Pharm.

[43] UNAIDS (2017). Global AIDS monitoring 2018: indicators for monitoring the 2016 United Nations political declaration on ending AIDS.

[44] World Health Organization, UNICEF, HMN, Countdown to 2015. Monitoring maternal, newborn and child health: understanding key progress indicators. Geneva: World Health Organization. p. 2011.

[45] Bryce J, Victora CG, Boerma T, Peters DH, Black RE. Evaluating the scale-up for maternal and child survival: A common framework. Int Health. 2011;3:139–46. 10.1016/j.inhe.2011.04.003 24038362

[46] Davis LM, Zalisk K, Herrera S, Prosnitz D, Coelho H, Yourkavitch J (2019). iCCM data quality: an approach to assessing iCCM reporting systems and data quality in 5 African countries. J Glob Health.

[47] Turinawe EB, Rwemisisi JT, Musinguzi LK, de Groot M, Muhangi D, de Vries DH (2015). Selection and performance of village health teams ( VHTs ) in Uganda : lessons from the natural helper model of health promotion.

[48] Atun R, de Jongh T, Secci FV, Ohiri K, Adeyi O (2009). Clearing the global health fog : a systematic review of the evidence on integration of health systems and targeted interventions (English).

[49] Laverack G, Wallerstein N (2001). Measuring community empowerment: a fresh look at organizational domains. Health Promot Int.

[50] Scott K, Beckham S, Gross M, Pariyo G, Rao K, Cometto G (2018). What do we know about community-based health worker programs? A systematic review of existing reviews on community health workers. Hum Resour Health.

[51] Jaskiewicz W, Tulenko K (2012). Increasing community health worker productivity and effectiveness: a review of the influence of the work environment. Hum Resour Health.

[52] PHCPI. Vital Signs Profiles. 2018. Retrieved from: https://improvingphc.org/vital-signs-profiles.

[53] Kuule Y, Dobson AE, Woldeyohannes D, Zolfo M, Najjemba R, Edwin BMR (2017). Community health volunteers in primary healthcare in rural Uganda: factors influencing performance. Front public Heal.

[54] Vallières F, Hyland P, McAuliffe E, Mahmud I, Tulloch O, Walker P (2018). A new tool to measure approaches to supervision from the perspective of community health workers: a prospective, longitudinal, validation study in seven countries. BMC Health Serv Res.

[55] Bhattacharyya K, LeBan K, Winch P, Tien M. Community health worker incentives and disincentives: how they affect motivation, retention, and sustainability. Arlington; 2001. Available from: https://pdf.usaid.gov/pdf_docs/PNACQ722.pdf

[56] Pallas SW, Minhas D, Perez-Escamilla R, Taylor L, Curry L, Bradley EH (2013). Community health workers in low- and middle-income countries: what do we know about scaling up and sustainability?. Am J Public Health.

[57] Naimoli JF, Perry HB, Townsend JW, Frymus DE, McCaffery JA (2015). Strategic partnering to improve community health worker programming and performance: features of a community-health system integrated approach. Hum Resour Health.

[58] Mohanan M, Vera-Hernández M, Das V (2015). The know-do gap in quality of health care for childhood diarrhea and pneumonia in rural India. JAMA Pediatr.

[59] Haines A, Sanders D, Lehmann U, Rowe AK, Lawn JE, Jan S (2007). Achieving child survival goals: potential contribution of community health workers. Lancet..

[60] MEASURE Evaluation (2017). Improving data quality in mobile community-based health information systems: guidelines for design and implementation.

[61] Dieleman M, Gerretsen B, van der Wilt GJ (2006). Human resource management interventions to improve health workers’ performance in low and middle income countries: a realist review. Heal Res Policy Syst..

[62] Bhatnagar A (2014). Determinants of motivation and job satisfaction among primary health workers: case studies from Nigeria and India.

[63] Dale EM (2014). Performance-based payments, provider motivation and quality of care in Afghanistan.

[64] Mpembeni RNM, Bhatnagar A, LeFevre A, Chitama D, Urassa DP, Kilewo C (2015). Motivation and satisfaction among community health workers in Morogoro region, Tanzania: nuanced needs and varied ambitions. Hum Resour Health.

[65] Glenton C, Colvin CJ, Carlsen B, Swartz A, Lewin S, Noyes J (2013). Barriers and facilitators to the implementation of lay health worker programmes to improve access to maternal and child health: qualitative evidence synthesis. Cochrane Database Syst Rev.

[66] Schaaf M, Fox J, Topp SM, Warthin C, Freedman LP, Robinson RS (2018). Community health workers and accountability: reflections from an international “think-in.”. Int J Equity Health.

[67] Bertrand J, Escudero G. Compendium of indicators for evaluating reproductive health programs: MEASURE Evaluation; 2002.

[68] MEASURE Evaluation (2013). Referral systems assessment and monitoring toolkit.

[69] James-Hawkins L, Peters C, VanderEnde K, Bardin L, Yount KM (2018). Women’s agency and its relationship to current contraceptive use in lower- and middle-income countries: a systematic review of the literature. Glob Public Health.

[70] Cyril S, Smith BJ, Renzaho AMN (2016). Systematic review of empowerment measures in health promotion. Health Promot Int.

[71] Muturi N, Nanamatsu Y, Mireku M, Regeru R, Okoth L, Doyle V, et al. Opening the black box: how to measure quality of household visits by community health workers in Kenya? Liverpool; 2018.

[72] Bova C, Fennie KP, Watrous E, Dieckhaus K, Williams AB (2006). The health care relationship (HCR) trust scale: development and psychometric Evaluation. Res Nurs Health.

[73] McCollum R, Gomez W, Theobald S, Taegtmeyer M (2016). How equitable are community health worker programmes and which programme features influence equity of community health worker services? A systematic review. BMC Public Health.

[74] Morgan R, Ayiasi RM, Barman D, Buzuzi S, Ssemugabo C, Ezumah N (2018). Gendered health systems: evidence from low- and middle-income countries. Heal Res Policy Syst.

[75] ICF. The DHS Wealth Index. n.d. Retrieved from: https://www.dhsprogram.com/topics/wealth-index/Wealth-Index-Construction.cfm.

[76] Cook KS, Hegtvedt KA (1983). Distributive justice, equity, and equality. Annu Rev Sociol.

[77] Ved R, Scott K, Gupta G, Ummer O, Singh S, Srivastava A (2019). How are gender inequalities facing India’s one million ASHAs being addressed? Policy origins and adaptations for the world’s largest all-female community health worker programme. Hum Resour Health.

[78] Frontline Health Workers Coalition (2018). Investing in the health workforce for women’s economic empowerment.

[79] Nkonki L, Tugendhaft A, Hofman K. A systematic review of economic evaluations of CHW interventions aimed at improving child health outcomes. Vol. 15, Human Resources for Health: BioMed Central Ltd; 2017. 10.1186/s12960-017-0192-5 PMC5331680 28245839

[80] Community Health Impact Coalition, UNICEF, USAID (2018). CHW AIM Updated Program Functionality Matrix for Optimzing Community Health Programs.

