# Supplementary material for: A conceptual framework for measuring community health workforce performance within primary health care systems
Source: Hum Resour Health. 2019 Nov 20;17:86. doi: 10.1186/s12960-019-0422-0 (PMC6868857; doi:10.1186/s12960-019-0422-0)
Supplement: Supplementary file 1 — Additional file 1. TAG Survey for Metrics Development: May 2018, 25 respondents. [file 12960_2019_422_MOESM1_ESM.docx]

Additional file 1: TAG Survey for Metrics Development: May 2018, 25 respondents

| Indicator | Domain important to measure?? (%) | | | Illustrative Indicator valuable?? (%) | | | | Indicator Status |
| --- | --- | --- | --- | --- | --- | --- | --- | --- |
|  | **Yes** | **No** | **Maybe** | **Yes** | **No** | **Unclear** | **Unsure** |  |
| **CHW DEVELOPMENT** |  |  |  |  |  |  |  |  |
| **Recruitment** | 72 | 4 | 24 |  |  |  |  |  |
| X: # of CHWs who have been selected in alignment with selection criteria |  |  |  | 64 | 44 | 24 | 8 | Retained as is |
| Y: # of CHWs who have been selected |  |  |  | 40 | 32 | 16 | 12 | Edited |
| Z: #/% of target communities/ populations that have an assigned CHW |  |  |  | 84 | 0 | 8 | 8 | Retained as is |
| **Training** | 68 | 8 | 24 |  |  |  |  |  |
| W: #/% of CHWs who have received initial training |  |  |  | 60 | 16 | 8 | 16 | Retained as is |
| X: #/% of CHWs who have received follow-up training |  |  |  | 56 | 12 | 16 | 16 | Edited |
| Y: #/% of CHWs who have completed the certification program |  |  |  | 84 | 0 | 8 | 8 | Retained as is |
| Z: #/% of CHWs who have received training on data entry and reporting |  |  |  | 36 | 24 | 12 | 28 | Omitted |
| **Incentives** | 80 | 4 | 16 |  |  |  |  |  |
| X: #/% of CHWs who have received their stipend in the last month |  |  |  | 76 | 8 | 8 | 8 | Retained as is |
| Y: #/% of CHWs who have received a specific non-financial incentive |  |  |  | 40 | 24 | 24 | 12 | Retained as is |
| **SUPPORT FROM COMMUNITY-BASED GROUPS** |  |  |  |  |  |  |  |  |
| **Support from community-based groups** | 60 | 4 | 36 |  |  |  |  |  |
| X: #/% of communities that have a community score card |  |  |  | 40 | 12 | 12 | 36 | Omitted |
| Y: # of workshops held to train CHW support groups |  |  |  | 20 | 28 | 4 | 48 | Omitted |
| Z: # of planning/ review meetings held at the level of the local government |  |  |  | 68 | 4 | 12 | 16 | Edited |
| **HEALTH SYSTEMS SUPPORT** |  |  |  |  |  |  |  |  |
| **Supervision** | 96 | 0 | 4 |  |  |  |  |  |
| X: #/% of supervisors trained in management and supervision of CHWs |  |  |  | 64 | 12 | 4 | 20 | Retained as is |
| Y: Average # of CHWs assigned to each supervisor |  |  |  | 60 | 12 | 4 | 24 | Edited |
| Z: # of health facilities trained in providing support to CHWs |  |  |  | 40 | 16 | 16 | 28 | Omitted |
| **Performance appraisal** | 84 | 8 | 8 |  |  |  |  |  |
| X: Average # of visits per supervisor to monitor/support CHW activities in the last month |  |  |  | 44 | 20 | 16 | 20 | Retained as is |
| Y: #/% of CHWs who received a supervisory visit in the last month |  |  |  | 76 | 8 | 4 | 12 | Edited |
| Z: Average # of supervisory contacts (in-person visits, phone calls, text messages, etc.) per CHW* | - | - | - | - | - | - | - | Added |
| **Data use** | 80 | 0 | 20 |  |  |  |  |  |
| X: #/% of CHWs/supervisors/health facility staff who have access to data, who report using the data to make decisions about their provision of services |  |  |  | 68 | 8 | 8 | 16 | Edited |
| Y: #/% of national/sub-national/facility meetings in which data (from standardized reporting platforms etc.) are discussed |  |  |  | 64 | 8 | 12 | 16 | Edited |
| Z: #/% of CHWs who have access to the client data they have collected (for follow-up) in the last 6 months |  |  |  | 44 | 12 | 12 | 32 | Retained as is |
| **CHW COMPETENCY** |  |  |  |  |  |  |  |  |
| **CHW knowledge** | 72 | 0 | 28 |  |  |  |  |  |
| X: #/% of CHWs who have passed knowledge tests |  |  |  | 64 | 0 | 12 | 24 | Edited |
| Y: #/% of CHWs who express that they feel confidence in their abilities to provide health education |  |  |  | 60 | 8 | 12 | 20 | Retained as is |
| Z: #/% of CHWs who express confidence in their abilities to deliver basic healthcare services |  |  |  | 64 | 16 | 8 | 12 | Retained as is |
| **Service delivery** | 96 | 0 | 4 |  |  |  |  |  |
| X: Average # of home visits made by CHWs in the last month |  |  |  | 72 | 8 | 4 | 16 | Edited |
| **Quality of services provided** | 96 | 0 | 4 |  |  |  |  |  |
| W: Average amount of time spent (per CHW) by the supervisor during the last supervisory visit |  |  |  | 28 | 36 | 16 | 20 | Omitted |
| X: % of CHWs who correctly identified the case/health problem |  |  |  | 76 | 8 | 8 | 8 | Edited |
| Y: % of CHWs who correctly addressed (treated) the identified health problem |  |  |  | 76 | 4 | 4 | 16 | Edited |
| Z: Average amount of time spent per household by the CHW during the last home-visit |  |  |  | 24 | 36 | 12 | 28 | Omitted |
| WW: #/% of CHWs with all the key stock commodities in the last reporting period* |  |  |  |  |  |  |  | Added |
| YY: Average time from onset of symptom to first contact with CHW* |  |  |  |  |  |  |  | Added |
| **Data reporting** | 72 | 8 | 20 |  |  |  |  |  |
| X: #/% of CHWs who submitted reports in the last month |  |  |  | 72 | 8 | 0 | 20 | Retained as is |
| Y: % of CHW reports submitted that were complete/did not have missing information |  |  |  | 80 | 4 | 0 | 16 | Retained as is |
| **CHW absenteeism** | 72 | 4 | 24 |  |  |  |  |  |
| X: #/% of CHWs who reported on their activities in the last month |  |  |  | 52 | 16 | 8 | 24 | Retained as is |
| Y: Average # of days that a CHW worked as a CHW in the last month |  |  |  | 36 | 16 | 20 | 28 | Edited |
| **CHW WELL-BEING** |  |  |  |  |  |  |  |  |
| **CHW motivation** | 72 | 4 | 24 |  |  |  |  |  |
| Would the development of a motivation scale/ adaptation of existing CHW motivation scales to different contexts be valuable? | 44 | 8 | 48 |  |  |  |  | Composite Metric Developed |
| **CHW job-satisfaction** | 68 | 4 | 28 |  |  |  |  |  |
| X: #/% of CHWs who expressed satisfaction with the community support they receive |  |  |  | 52 | 12 | 8 | 28 | Retained as is |
| Y: #/% of CHWs who expressed satisfaction with the support they receive from health facility staff |  |  |  | 64 | 8 | 4 | 24 | Retained as is |
| **Attrition/ Retention** | 96 | 4 | 0 |  |  |  |  |  |
| X: % of CHWs who have reported on their activities in the last 6 months |  |  |  | 48 | 12 | 24 | 16 | Edited |
| **ACCESS TO SERVICES** |  |  |  |  |  |  |  |  |
| **Use of services** | 88 | 0 | 12 |  |  |  |  |  |
| X: Percentage of pregnant women who were seen by a CHW within 2 days of home-birth** |  |  |  | 80 | 0 | 4 | 16 | Omitted |
| Y: #/% of households who received at least one visit by a CHW in the last 3 months |  |  |  | 76 | 0 | 8 | 16 | Retained as is |
| Z: Percentage of all births that took place in a health facility accompanied by a CHW |  |  |  | 44 | 16 | 8 | 32 | Omitted |
| **Knowledge of service availability** | 68 | 28 | 4 |  |  |  |  |  |
| X: #/% of community members that know the name of the community CHWs* | - | - | - | - | - | - | - | Added |
| Y: #/% of community members who can name at least 3 services that the CHW provides* | - | - | - | - | - | - | - | Added |
| **Referral/ counter-referral** | 88 | 0 | 12 |  |  |  |  |  |
| X: % of individuals referred to the health facility from the total number of visits (by reasons for referral) |  |  |  | 64 | 8 | 8 | 20 | Edited |
| Y: % of clients that completed the referral (referral completion) |  |  |  | 76 | 4 | 8 | 12 | Edited |
| Z: % of referred clients seen at receiving service (health facility) that is seen back at referring service (CHW) with complete counter-referral information (counter-referral) |  |  |  | 56 | 8 | 20 | 16 | Retained as is |
| XX: Average # of referrals made per CHW in the last month* | - | - | - | - | - | - | - | Added |
| **COMMUNITY-CENTERED CARE** |  |  |  |  |  |  |  |  |
| **Empowerment** | 64 | 8 | 28 |  |  |  |  | Composite Metric Developed |
| **Experience of services** | 92 | 0 | 8 |  |  |  |  | Edited |
| X: #/% of women/households who express satisfaction with the services they received from the CHW in the last 6 months |  |  |  | 72 | 20 | 0 | 8 | Edited |
| Y: #/% of women who report that in their interaction with the CHW they felt humiliated or disrespected (scale 1-5) |  |  |  | 68 | 16 | 4 | 12 | Retained as is |
| **Economic evaluation** | - | - | - | - | - | - | - | No extant or routine indicators identified |
| **Credibility/ trust of CHW** | 76 | 16 | 8 |  |  |  |  |  |
| X: #/% of women/clients who report they trust the health information provided by the CHW |  |  |  | 100 | 0 | 0 | 0 | Retained as is |
| Y: #/% of women/clients who report they trust the treatment services provided by the CHW |  |  |  | 100 | 0 | 0 | 0 | Retained as is |

*This metric was added later in the indicator development process in consultation with measurement experts, after the TAG survey was conducted and results finalized.

**Despite the agreement on the importance of this metric, it was excluded from the generic set of indicators presented in Table 2 based on the decision to exclude any health-area specific indicators.
